# E-health literacy levels of multiple sclerosis patients in Lebanon

**DOI:** 10.1371/journal.pone.0335084

**Published:** 2025-10-31

**Authors:** Maria Rita Lteif, Joumana Kalot, Mahmoud El Jaouni, Samia J. Khoury

**Affiliations:** 1 Department of Health Promotion and Community Health, American University of Beirut, Beirut, Lebanon; 2 Center for Public Health Practice & Department of Health Promotion and Community Health, Faculty of Health Sciences, American University of Beirut, Beirut, Lebanon; 3 Department of Epidemiology and Population Health, American University of Beirut, Beirut, Lebanon; 4 The Nehme and Therese Tohme Multiple Sclerosis Center, American University of Beirut, Beirut, Lebanon; The University of Warwick, UNITED KINGDOM OF GREAT BRITAIN AND NORTHERN IRELAND

## Abstract

**Background:**

Having high levels of e-health literacy has been shown to positively influence health-promoting behaviors by empowering patients with greater self-efficacy and improving their health management. Multiple Sclerosis (MS) in Lebanon presents a unique case for studying e-health literacy due to several socio-economic and healthcare-specific challenges faced by this population.

**Aim:**

This study aimed to evaluate the e-health literacy levels of MS patients in Lebanon and identify the barriers they face when accessing health-related information online.

**Methods:**

This study employed a mixed-methods approach, combining an online structured survey with qualitative in-depth interviews. The eHealth Literacy Scale (eHEALS) was incorporated to quantify participants’ e-health literacy levels, selected for its well-established reliability and validity, as well as its availability in both English and Arabic. Additionally, the Lily Model was used to guide various phases of the study, providing a conceptual framework for understanding and interpreting e-health literacy.

**Results:**

Among participants, 45 (31.5%) had limited eHEALS scores while 98 (68.5%) had sufficient eHEALS scores, indicating an overall sufficient e-health literacy among MS patients. However, qualitative findings revealed previously unidentified challenges, including limited confidence in assessing the credibility of online content, emotional distress triggered by exaggerated MS information online, and high reliance on general search engines and social media platforms. Participants expressed lack of access to trusted disease-specific information and voiced a need for professional guidance and well-established peer support.

**Conclusion:**

These findings call for targeted health promotion interventions to enhance this population’s e-health literacy levels. It is important to equip MS patients from the point of diagnosis with the necessary skills and self-efficacy to effectively utilize online health resources and enhance their disease management journey. This calls for coordinated efforts from well-established MS patient groups, healthcare providers, academic and private institutions to support and empower this population in navigating the digital health landscape.

## Introduction

### Concept and framework of e-health literacy

E-health literacy is defined as the ability to seek, find, understand, and assess health information from electronic sources and apply the knowledge gained to address or manage a health problem [1]. The concept of E-health literacy has also shown to be essential in improving healthcare delivery and quality of care; with patients having high e-health literacy levels being more empowered to take control over their health and make proper decisions in managing their disease [2]. In other words, having high levels of e-health literacy can promote online health-seeking behavior and enhance self-care agency [3]. An individual’s level of E-health literacy changes as new technologies are introduced and when personal, social, and environmental contexts evolve [4]. The Lily model, developed by Norman and Skinner, identifies six core skills that can either hinder or enable an individual’s ability to engage with digital health information: traditional literacy (reading and writing), health literacy (understanding and applying health information), information literacy (finding and evaluating information), scientific literacy (interpreting scientific concepts), media literacy (analyzing media content), and computer literacy (using digital technology effectively) [4,5]. This model is based in part on the social cognitive theory and in part on the self-efficacy theory, both of which help explain how individuals learn and develop confidence in using technology for health-related outcomes [1].

### Global Evidence on E-Health Literacy Across Populations

E-health literacy has been globally studied among university students, older adults, and general internet users [6–9]. These studies assessed individuals’ perceived ability to use online information for health purposes. A study which examined the e-health literacy levels of undergraduate nursing students in the U.S. and South Korea identified a gap in how and where they seek reliable online health resources. Meaning, students lacked confidence in using online information for health-related decisions and faced challenges in assessing the quality of internet-based health resources [9]. In older adults, technological and physical limitations were perceived as the primary barriers to their confidence in using online health information [6]. As a result, they continue to rely on traditional sources like physicians and television for health information, valuing personal interaction more than online resources [10]. Meanwhile, digital health interventions in Germany are likely to benefit individuals with lower educational and social status, as they exhibited less confidence in their ability to seek health information online [11]. The literature thus highlights global variations in e-health literacy levels across different sub-populations, with common challenges related to trust in the credibility of online information.

### E-Health Literacy in the Arab Region

In the Arab region, online health seeking behavior was assessed across different sub-populations in the Kingdom of Saudi Arabia (KSA), the United Arab Emirates (UAE), Egypt, Iran, Kuwait, and Lebanon. For example, a recent study conducted among the general population in the KSA indicated that a significant majority of participants find it easy to search for health-related information online, however, assessing the reliability of the information was challenging. This once again highlights the critical need for specialized educational initiatives on digital health literacy in the country [12]. The Kuwaiti population was found to use the internet as a primary source of health information, followed by physicians, and social media platforms [13]. These findings suggest a shift in trust and reliance on information sources, with a changing role of physicians and an increased trust in the internet [13]. Similarly, in Iran, undergraduate students were found to rely primarily on the internet for mental health information; however, their trust in this online information is low, with greater trust placed in healthcare professionals [14]. Such results can be explained by the ease of access to the internet and the advances in technology, making online health searches more accessible [14].

### E-Health Literacy and Chronic Disease Management

Despite this general focus, limited research has examined the impact of e-health literacy on individuals with chronic diseases and their ability to access high-quality online information for actively managing their health [15]. Among adult patients with diabetes in KSA, those who sought health information online were more likely to regularly self-monitor their blood glucose, take appropriate actions for hyperglycemia, and adopt nonpharmacological management strategies [16]. Participants however still considered physicians and television as primary sources of health-related information, even among those who seek information online [16]. Such studies have solely relied on subjective perceptions of e-health literacy and did not comprehensively evaluate the various challenges and facilitators related to accessing health information online and improving disease management.

### Multiple Sclerosis and E-health Literacy

One specific population that remains understudied in the context of e-health literacy and chronic diseases is MS patients. This is particularly concerning given that MS patients frequently engage in online health-related searches at the time of diagnosis [17,18]. MS is an immune-mediated inflammatory demyelinating disorder of the Central Nervous System (CNS), with clinical features that vary and may include symptoms of cerebral, brainstem, or spinal cord dysfunction [19]. This condition is most diagnosed between the ages of 20 and 40, with a higher prevalence among females (ratio 2.5:1) [20]. Although the exact cause of MS is yet to be elucidated, strong evidence links its development to Epstein–Barr virus infection, limited sunlight exposure, vitamin D deficiency, smoking, and genetic predisposition [19]. There is no cure for MS to date, however several disease modifying therapies are approved aimed at controlling disease progression, reducing the frequency of relapses, and managing symptoms [21]. MS patients, like patients with other chronic conditions, increasingly search for health information on the Internet to make decisions related to their health [18]. This information-seeking behavior is essential as it helps them understand their condition, solve problems, and maintain control over their lives [22]. However, MS remains poorly understood by the general public and among MS patients themselves, who know very little about their condition upon diagnosis and begin an active search for information [17]. This is particularly concerning given the global increase in MS prevalence from 2.3 million in 2013 to 2.9 million in 2023, alongside limited knowledge of the condition [23].

Multiple sclerosis patients increasingly rely on digital platforms for health information, yet they face significant challenges in verifying the accuracy and credibility of online content. Physicians remain the most trusted source of information due to the prevalence of misleading or incorrect information encountered online, leading some patients to abandon the internet for health information seeking [24]. A study conducted by the North American Research Committee on Multiple Sclerosis found that the internet was the first source of information for MS patients (59.23%), followed by health care providers (15.28%), and the National Multiple Sclerosis Society (13.04%) however, only 22.63% reported a lot of trust in the Internet [25]. The range of information sought by people with MS varies based on their needs, which are likely to change over the course of the disease, including an increase in the use of social networking platforms and support groups for the direct exchange of personal health information [25]. A qualitative study in Australia among MS patients found that these patients experience difficulties in finding and applying information for their specific needs, with a few participants expressing a fear of finding negative information online [18]. Additionally, skepticism regarding the Internet as a source of quality information was common, with most participants expressing a desire to discuss their Internet searches with doctors [18].

### E-Health Literacy in Lebanon’s Context: A Focus on MS Patients

Evaluating the e-health literacy levels of MS patients in Lebanon remains unexplored. On October 2019, Lebanon began to face an economic collapse, the US dollar became very scarce, subsidization of medical care as well as many other services were slowly terminating, and COVID-19 exhausted the country’s health care systems [8]. With many unable to secure basic services in the country, there is still no published information on whether patients are resorting to digital health to compensate for the difficulties in accessing healthcare. Among the populations most affected by the crisis are MS patients, who have struggled to access their medications. There are still no studies that aimed to understand their e-health literacy levels to improve and help meet their needs. Without a clear understanding of this, MS patients may be at risk of misinformation which can negatively impact their health. While online information has the potential to empower individuals to manage their health effectively, it also poses risks that stem from the quality of information available and the varying levels of e-health literacy across different domains. By identifying the barriers that hinder access to reliable and accurate information, targeted interventions to improve MS patients’ e-health literacy in Lebanon can be better tailored to this population, thus empowering them to make better decisions about their health. This study aimed to measure the e-health literacy levels of MS patients in Lebanon and identify the barriers they face in accessing health related information online.

## Materials and methods

### Study design and data collection tools

This study employed a mixed-methods approach, combining an online structured survey with qualitative in-depth interviews (IDIs) to ensure a comprehensive understanding of e-health literacy among MS patients in Lebanon.

The survey was developed using LimeSurvey and disseminated online, as MS patients are dispersed across Lebanon, making in-person data collection challenging. The questions were developed using the six core elements of Norman and Skinner’s e-health literacy model, the eHEALS 5-point Likert scale, in addition to sociodemographic information, and questions related to the ongoing 2019 crisis in Lebanon [1]. The eHEALS questionnaire was selected due to its robust reliability, validity, and its availability in both English and Arabic [26]. Given that the majority of participants were Arabic speakers, the eHEALS translation used in this study was sourced from a prospective psychometric evaluation conducted among an Arabic-speaking population, which confirmed its validity [26]. Total scores range from 8 to 40, with higher scores indicating greater self-perceived e-health literacy. Scores were further dived into the threshold values of inadequate (8–20 points), problematic (21–26 points), and sufficient (27–40 points), as well as categorized into limited (inadequate + problematic = 8–26 points) and sufficient (27–40 points) [27].

It was key to also add a qualitative component to this study since the eHEALS questionnaire is not without its limitations, with participants known to answer questions based on their own preferences and believes, not their actual capabilities. An IDI guide was developed to get a deeper understanding of participants’ true capabilities, the specific barriers they face in accessing high quality health related information online, and how this affects their ability to make health informed decisions. An initial interview guide (S1 File) was developed and subsequently refined into a final version after the first three interviews by incorporating probing questions and rephrasing questions to enhance clarity and participant understanding (S2 File).

All IDIs were conducted online either via Zoom or WhatsApp by the PI who was both trained and experienced in qualitative studies. To establish rapport, the interviewer began each interview by introducing herself and sharing her professional background and experience to build trust and create a comfortable environment for open dialogue.

### Ethics statement

Approval for this study was obtained from the Institutional Review Board (IRB) of the American University of Beirut under protocol number SBS-2023–0328.

As per IRB approval, participants provided written electronic informed consent to participate in this study through LimeSurvey using a two-step process. At the beginning of the survey, after reviewing the consent form detailing the study’s purpose, procedures, confidentiality, and voluntary participation, individuals were asked to indicate their consent by selecting “Yes” or “No. Similarly, at the end of the survey, they were asked again to provide consent for participation in an IDI by responding “Yes” or “No”.

### Eligibility criteria

Participants were eligible to participate in this study if they were multiple sclerosis patients between the ages of 18 and 65, reside in Lebanon, and actively use the internet.

Individuals were excluded from the study if they had visual impairments, cognitive impairments, or do not use the internet as these participants would face difficulties in accessing the survey or participating actively in the in-depth interviews.

### Sampling and recruitment

MS patients were purposively sampled from two well-established patient advocacy groups (patient led NGOs) in Lebanon and a specialized MS center in Beirut. This approach was the most appropriate, as no formal sampling frame or national database exists for MS patients in the country.

The survey was disseminated online on February 13^th^ 2024 through the advocacy groups’ WhatsApp channels (their primary communication platform with MS patients) and was closed on August 1^st^ 2024. Additionally, an invitation flyer with a QR code linked to the survey was placed in the waiting and treatment rooms of the selected MS center in Beirut. To maximize reach, the same information was also posted on the MS center’s Facebook page.

After half of the sample size was attained, participants were purposively sampled for an IDI depending on their survey responses and interest to participate. Participants were purposively sampled to take part in the IDIs based on their computed level of e-health literacy from the survey, the challenges they reported, and any notable preferences or concerns that required further exploration.

### Analysis

Statistical analysis was conducted using STATA version 17 and a p-value of less than 0.05 was considered statistically significant. Descriptive analysis was used to summarize the sample characteristics, with frequencies and percentages reported for categorical variables, and means, standard deviations, median, and interquartile range for continuous variables. Bivariate analyses (t-test, chi-squared test, or Fisher’s exact test) were performed to examine differences between survey completers and non-completers. Bivariate tests were also used to explore associations between sociodemographic characteristics and e-health literacy levels (limited vs. sufficient).

Participants were categorized into two groups based on their eHEALS score: Limited Group (eHEALS score between 8–26 points) and Sufficient Group (eHEALS score between 27–40). Additionally, for the purpose of analysis, sociodemographic categories were grouped together based on frequencies and order to be able to carry out bivariate analysis using chi squared test.

For the qualitative analysis, all IDIs were transcribed verbatim in Arabic and only quotes used in the results were translated to English. Thematic analysis was done following Braun and Clarke’s 6 phase guide of (1) familiarization with the data, (2) coding, (3) searching for themes/patterns, (4) reviewing themes, (5) defining and naming themes, and (6) developing the write up (33). The original coding matrix included 17 sub-themes that were generated from Steps 1 and 2 listed above. Examples of these subthemes include: “Where to search for information online”, “Internet use during crisis”, “How to check for credibility” and “Challenges”. These subthemes were then reviewed and defined into 8 overarching themes and presented in Table 10 based on the subsequent steps of Braun and Clarke’s guide.

## Results

### Sample characteristics and quantitative results

A total of 147 participants completed the quantitative survey during the recruitment window (February 13^th^ 2024 to August 1^st^ 2024). Of these, 4 were ineligible to participate leading to their removal from the analysis and a final sample size of 143 participants was achieved. Although initial sample size calculations indicated a higher target, several challenges encountered during data collection, such as the voluntary nature of questions, a high proportion of incomplete responses, and an overall low response rate, limited our ability to reach the intended sample size. To mitigate this limitation and enhance the depth of the findings, qualitative interviews were incorporated as a complementary method. Among the final sample, 127 participants completed all domains of the eHEALS scale (completers), while 16 participants left some domains unanswered (non-completers), which is not uncommon in online self-filled surveys.

Table 1 summarizes the sociodemographic characteristics of our sample and Table 2 illustrates their internet behaviors and habits. The mean age of participants was 39.69 years, and age was found to be normally distributed according to the Kolmogorov–Smirnov test (p = 0.161). The sample was predominantly female (66.43%), of Lebanese nationality (95.77%), with 72.54% having an educational level above school (Bachelor’s, Master’s, or PhD). Additionally, 50.7% were employed and 61.54% were married. The survey was completed in both English (53.85%) and Arabic (46.16%), with almost all respondents having internet at home and use it on their own. When it comes to using the internet for information on MS, 26.06% said that they use it frequently, 53.52% use it sometimes, and 20.42% never or seldomly use the internet for MS.

**Table 1:** Sociodemographic characteristics of the total sample of participants (N = 143).

Variables	n (%) | Mean ± SD
**Age**	39.69 ± 10.46
**Gender**	
Female	95 (66.43%)
Male	48 (33.57%)
**Nationality**	
Lebanese	136 (95.77%)
Non-Lebanese	6 (4.23%)
**Education Level**	
Basic	8 (5.63%)
School Level	31 (21.83%)
Higher	103 (72.54%)
**Occupational Status**	
Employed	72 (50.70%)
Unemployed	70 (49.30%)
**Marital Status**	
Married	88 (61.54%)
Not Married	55 (38.46%)
**Language of Survey**	
Arabic	66 (46.15%)
English	77 (53.85%)

N= total sample, n= frequency, %= percentage, SD= Standard Deviation

**Table 2:** Internet use and behavior among participants.

Variables	n	%
**Home internet**		
Yes	138	96.5
No	5	3.5
**Internet use**		
Yes, on my own	137	96.48
Yes, with the help of others	5	3.52
**Hours spent per day on the internet**		
< 1 hour	8	5.59
1–3 hours	58	40.56
3–5 hours	32	22.38
> 5 hours	45	31.47
**Frequency of using the internet for MS**		
Never	7	4.93
Seldom	22	15.49
Sometimes	76	53.52
Frequently	37	26.06

Completers and non-completers of the eHEALS were compared using independent t-tests (age), Chi-square tests, or Fisher’s exact tests (Table 3). No statistically significant differences were observed between the two groups across most variables, including age, gender, nationality, occupational status, marital status, and the language in which the survey was completed. Educational level was borderline significant with a P-value of 0.05. This suggests a potential association between higher education and survey completion, as 75% of individuals who completed the survey had attained higher education, compared to only 56% among non-completers.

**Table 3:** Differences Between eHEALS Completers and Non-completers.

Variables	Completers n (%)	Non-completers n (%)	P-value
**Age** (Mean ±SD)	39.36 ± 10.56	42.85 ± 9.25	0.254
**Gender**			0.724
Female	85 (66.93)	10 (62.50)	
Male	42 (33.07)	6 (37.50)	
**Nationality**			0.519
Lebanese	121 (96.03)	15 (93.75)	
Non-Lebanese	5 (3.97)	1 (6.25)	
**Education Level**			0.050
Basic	5 (3.97)	3 (18.75)	
School-level	27 (21.43)	4 (25.00)	
Higher	94 (74.60)	9 (56.25)	
**Occupational Status**			0.555
Employed	65 (51.59)	7 (43.75)	
Unemployed	61 (48.41)	9 (56.25)	
**Marital Status**			0.529
Married	77 (60.63)	11 (68.75)	
Not Married	50 (39.37)	5 (31.25)	
**Language of Survey**			0.390
Arabic	57 (44.88)	9 (56.25)	
English	70 (55.12)	7 (43.75)	

*P-value < 0.05

Age was compared using an independent t-test; categorical variables were compared using Chi-square test, with Fisher’s exact test applied when more than 20% of expected cell counts were <5

**Fig 1:**
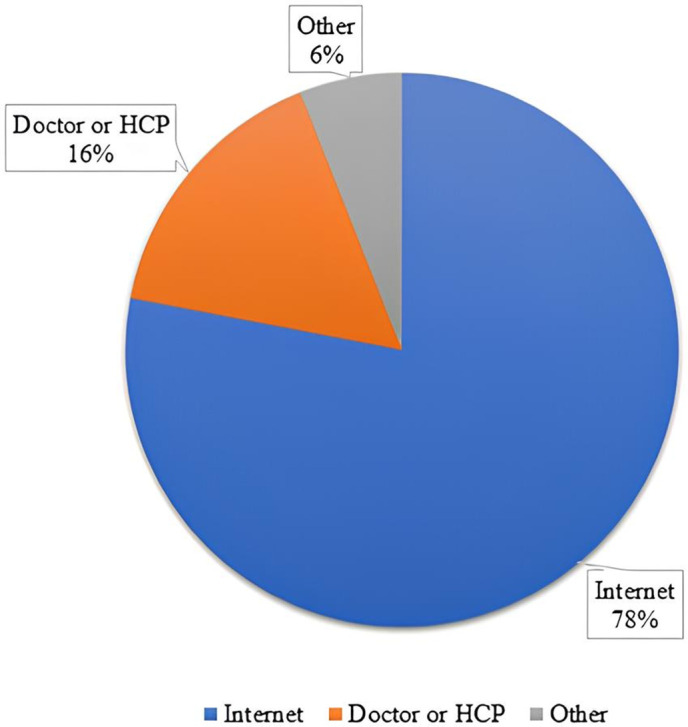
Participants’ most recently accessed resource for health information.

**Fig 2:**
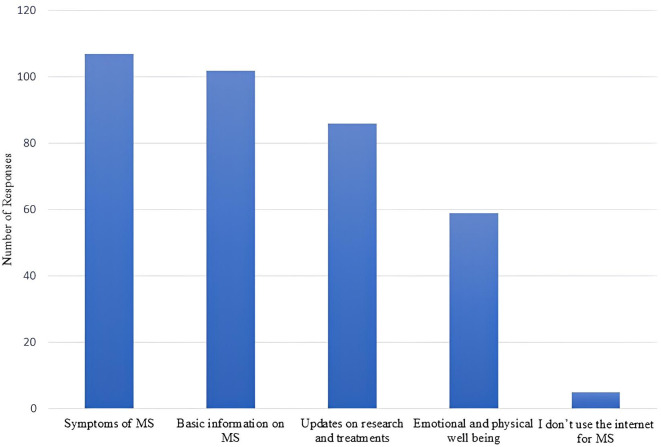
Most common reasons behind internet search on MS.

Looking at the variables related to e-health literacy and internet behaviors, when asked what was the first resource participants resorted to the last time they were looking for health information, 78% said they used the internet, 16% said they relied on their doctor or health care provider (HCP), and 6% reported other resources such as family, friends/coworkers, and books (Fig 1). Participants mainly use the internet for MS to look for information related to symptoms, basic information on MS, updates on research and treatment, and information on emotional and physical wellbeing (Fig 2). For those who don’t use the internet to look for information on MS, which make a very small proportion of our sample, they identified the following reasons behind this decision: a preference to rely on HCPs, internet searches cause anxiety and stress, and a belief that they already possess sufficient information about MS.

**Fig 3:**
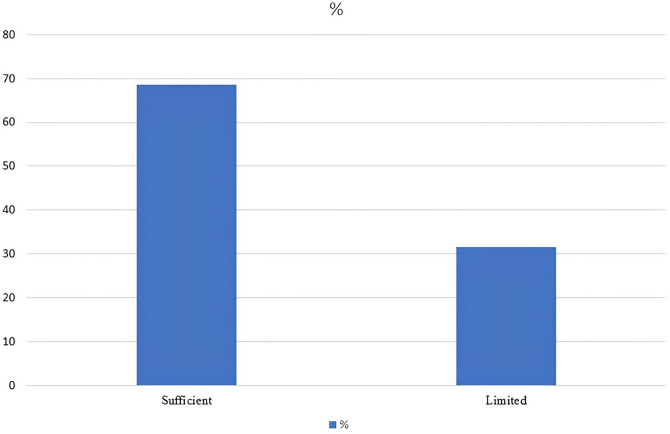
Percentage of participants categorized as ‘limited’ and ‘sufficient’ based on eHEALS scores.

The distribution of the total eHEALS scores was tested using the Kolmogorov–Smirnov test, which revealed a significant deviation from normality (p-value = 0.00). This non-normal distribution justified reporting both means (SD) and medians (IQR) to provide a comprehensive view of the results (Table 4). The mean total score was 28.57 (±5.30), and the median was 30, with an interquartile range of 25–32, indicating a moderate level of e-Health literacy among respondents. When participants were categorized based on their eHEALS scores, 68.5% had sufficient e-health literacy and 31.5% had limited e-health literacy (Fig 3). The narrow IQR (7-point spread) suggests a relatively consistent distribution of responses within the middle 50% of the participants. The per item response to the eHEALS is presented in S1 Table as n (%).

**Table 4:** Participant’s eHealth literacy scale (eHEALS) results.

eHEALS items	Mean ± SD	Median (Interquartile Range)
**I know what health resources are available on the Internet.**	3.37 ± 0.85	3 (3–4)
**I know where to find helpful health resources on the Internet.**	3.59 ± 0.82	4 (3–4)
**I know how to find helpful health resources on the Internet.**	3.60 ± 0.78	4 (3–4)
**I know how to use the Internet to answer my health questions.**	3.76 ± 0.75	4 (4–4)
**I know how to use the health information I find on the Internet to help me.**	3.78 ± 0.71	4 (4–4)
**I have the skills I need to evaluate the health resources I find on the Internet.**	3.54 ± 0.85	4 (3–4)
**I can tell high-quality health resources from low-quality health resources on the Internet.**	3.56 ± 0.89	4 (3–4)
**I feel confident in using information from the Internet to make health decisions.**	3.38 ± 1.01	4 (3–4)
**Total Score**	28.57 ± 5.30	30 (25–32)

Comparing individuals with limited and sufficient eHEALS scores is essential to explore potential associations between digital health literacy and participants’ demographic characteristics as well as their perceptions and behaviors related to online health information. As presented in Table 5, a statistically significant difference was identified in two aspects: the perceived importance of accessing health information online (p = 0.010) and confidence in the ability to identify potentially biased or misleading health information online (p = 0.001). Specifically, a higher proportion of individuals in the sufficient group (81.82%) perceived the internet as extremely important for accessing health information online compared to those in the limited group (18.18%). Similarly, 80.90% of individuals in the sufficient group reported confidence in their ability to identify potentially biased or misleading health information online while only 19.10% of those in the limited group expressed the same confidence. No other statistically significant differences were observed between participants with limited and sufficient eHEALS scores.

**Table 5:** Comparison of sociodemographic characteristics of those who have limited and sufficient eHEALS scores.

Variables	n (%) | Mean ± SD		P-value
	Limited eHEALS	Sufficient eHEALS	
Age (years)	41.18 ± 10.62	38.97 ± 10.36	0.251
Age Categories (9 missing values)			0.388
Young Adults (18-39)	19 (29.23)	46 (70.77)	
Older Adults (40-65)	25 (36.23)	44 (63.77)	
Gender			0.062
Female	25 (26.32)	70 (73.68)	
Male	20 (41.67)	28 (58.33)	
Nationality			0.665
Lebanese	44 (32.35)	92 (67.65)	
Non-Lebanese	1 (16.67)	5 (83.33)	
Education Level			0.916
Basic	2 (25.00)	6 (75.00)	
School Level	10 (32.26)	21 (67.74)	
Higher	33 (32.04)	70 (67.96)	
Occupational Status			0.670
Employed	24 (33.33)	48 (66.67)	
Unemployed	21 (30.00)	49 (70.00)	
Marital Status			0.909
Married	28 (31.82)	60 (68.18)	
Not Married	17 (30.91)	38 (69.09)	
Language of Survey			0.657
Arabic	22 (33.33)	44 (66.67)	
English	23 (29.87)	54 (70.13)	
Frequency of using the internet for MS			0.960
Never	2 (28.57)	5 (71.43)	
Seldom	8 (36.36)	14 (63.64)	
Sometimes	24 (31.58)	52 (68.42)	
Frequently	11 (29.73)	26 (70.27)	
Perceived Importance in Accessing Health Information Online			0.010*
Extremely important	12 (18.18)	54 (81.82)	
Moderately important	25 (43.86)	32 (56.14)	
Neutral	4 (40.00)	6 (60.00)	
Slightly important	3 (60.00)	2 (40.00)	
Not important at all	1 (20.00)	4 (80.00)	
Reported Challenges in accessing health information online related to MS			0.306
Yes	19 (37.25)	32 (62.75)	
No	26 (28.89)	64 (71.11)	
Confidence in ability to identify potentially biased or misleading health information online			0.001*
Yes	17 (19.10)	72 (80.90)	
No	27 (51.92)	25 (48.08)	

*P-value < 0.05

Age was compared using an independent t-test; categorical variables were compared using Chi-square test, with Fisher’s exact test applied when more than 20% of expected cell counts were <5

Bivariate analysis was performed between the sociodemographic variables (age, gender, and occupational status) and the four variables: language of completed survey, hours spent per day on the internet, frequency of using the internet for MS, and opinion on the usefulness of the internet in making health decisions (Tables 6–8). Most associations were found to be statistically insignificant while two significant ones emerged. The first was between occupational status and the language in which the survey was completed (P-value = 0.004). This indicates that employed participants were more likely to complete the survey in English, while unemployed participants were more likely to complete it in Arabic. The second significant association was between age and the number of hours spent per day on the internet (P-value = 0.001) showing that as age increases, the number of hours spent online decreases.

**Table 6:** Bivariate analysis of sociodemographic variables and survey language.

Variables	n (%) | Mean ± SD		P-value
	Language of Completed Survey		
	Arabic	English	
**Age**	40.03 ± 10.62	39.41 ± 10.38	0.733
**Gender**			0.085
Female	39 (41.05)	56 (58.95)	
Male	27 (56.25)	21 (43.75)	
**Occupational Status**			0.004*
Employed	25 (34.72)	47 (65.28)	
Unemployed	41 (58.57)	29 (41.43)	

*P-value < 0.05

Age was compared using an independent t-test; categorical variables were compared using Chi-square test, with Fisher’s exact test applied when more than 20% of expected cell counts were <5

**Table 7:** Bivariate analysis of sociodemographic variables and internet use patterns.

Variables	n (%) | Mean ± SD				P-value
	Hours Spent Per Day on the Internet				
	< 1 hour	1 – 3 hours	3 – 5 hours	> 5 hours	
**Age**	48.17 ± 8.77	42.31 ± 9.31	39.31 ± 10.39	35.52 ± 10.65	0.001*
**Gender**					0.706
Female	6 (6.32)	41 (43.16)	21 (22.11)	27 (28.42)	
Male	2 (4.17)	17 (35.42)	11 (22.92)	18 (37.50)	
**Occupational Status**					0.709
Employed	4 (5.56)	27 (37.50)	15 (20.83)	26 (36.11)	
Unemployed	4 (5.71)	30 (42.86)	17 (24.29)	19 (27.14)	
**Variables**	**Frequency of Using the Internet for MS**				**P-value**
	Never	Seldom	Sometimes	Frequently	
**Age**	41.71 ± 6.61	35.60 ± 9.43	39.01 ± 10.29	43.12 ± 11.41	0.066
**Gender**					0.346
Female	4 (4.21)	14 (14.74)	48 (50.53)	29 (30.53)	
Male	3 (6.38)	8 (17.02)	28 (59.57)	8 (17.02)	
**Occupational Status**					0.457
Employed	4 (5.56)	9 (12.50)	37 (51.39)	22 (30.56)	
Unemployed	2 (2.90)	13 (18.84)	39 (56.52)	15 (21.74)	

*P-value < 0.05

Age was compared using an independent t-test; categorical variables were compared using Chi-square test, with Fisher’s exact test applied when more than 20% of expected cell counts were <5

**Table 8:** Bivariate analysis of sociodemographic variables and perceived usefulness in health decision-making.

Variables	n (%) | Mean ± SD			P-value
	Opinion on the Usefulness of the Internet in Making Health Decisions			
	Not Useful	Unsure	Useful	
**Age**	40.18 ± 10.23	40.05 ± 11.11	39.46 ± 10.29	0.947
**Gender**				0.922
Female	8 (8.42)	28 (29.47)	59 (62.11)	
Male	4 (8.33)	12 (25.00)	32 (66.67)	
**Occupational Status**				0.620
Employed	5 (6.94)	22 (30.56)	45 (62.50)	
Unemployed	7 (10.00)	17 (24.29)	46 (65.71)	

*P-value < 0.05

Age was compared using an independent t-test; categorical variables were compared using Chi-square test, with Fisher’s exact test applied when more than 20% of expected cell counts were <5

**Fig 4:**
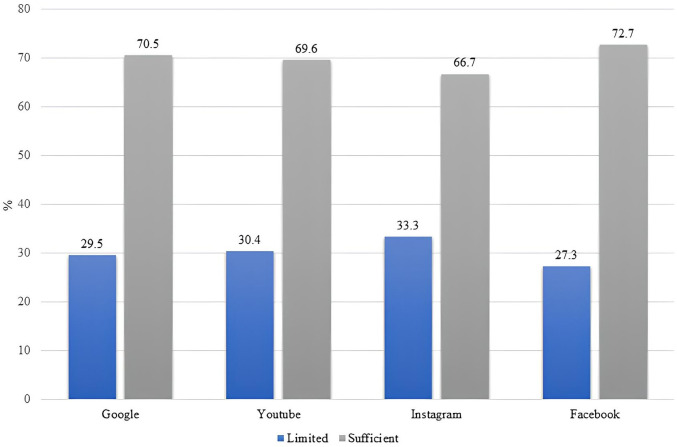
Comparison of the different platforms used in limited and sufficient eHEALS groups.

Fig 4 illustrates the prevalence of using various online platforms to seek health information among individuals with limited and sufficient e-health literacy. Google and Facebook were the most commonly used search engines online, followed by YouTube and Instagram. Snapchat and TikTok are rarely used for seeking health information online among participants whether they had limited or sufficient e-health literacy.

**Fig 5:**
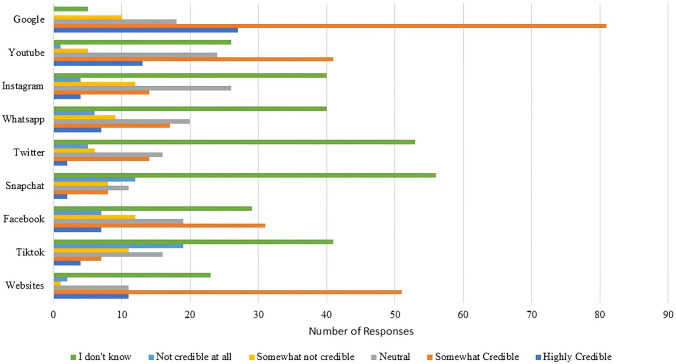
Participants’ ratings of the credibility of various online platforms.

Survey results also identified several challenges and preferences when it comes to online health seeking behavior among participants. More than 60% of respondents said that they did not experience any challenges in accessing health information online related to MS. While those who did report challenges identified the following reasons in decreasing order: information overload, lack of trust and credibility in online information, difficulties in understanding medical terminology, lack of MS-specific information, absence of Arabic resources, conflicting information, and non-user-friendly websites. Almost 40% of survey respondents said that they do not know how to identify potentially biased or misleading health advertisements or news stories posted online. Additionally, almost all participants said that they don’t know whether the platforms they use are credible or not when it comes to seeking health information online, especially for Snapchat and Twitter (Fig 5).

**Fig 6:**
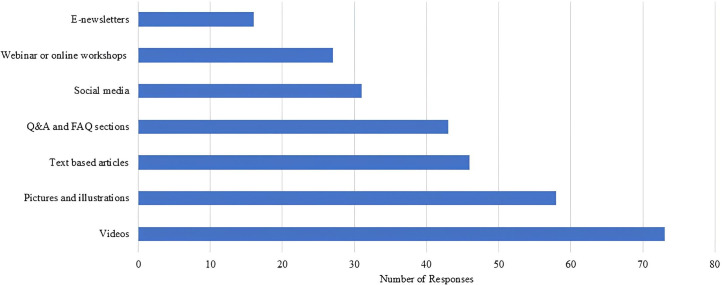
Most preferred formats to accessing online health information.

Regarding participants’ preferences of online health information, visual materials such as videos, pictures, and illustrations were the most preferred. These were followed by written formats including text-based articles, FAQ sections, and social media posts. Webinars or online workshops and E-newsletters were the least preferred formats among the listed options (Fig 6).

**Fig 7:**
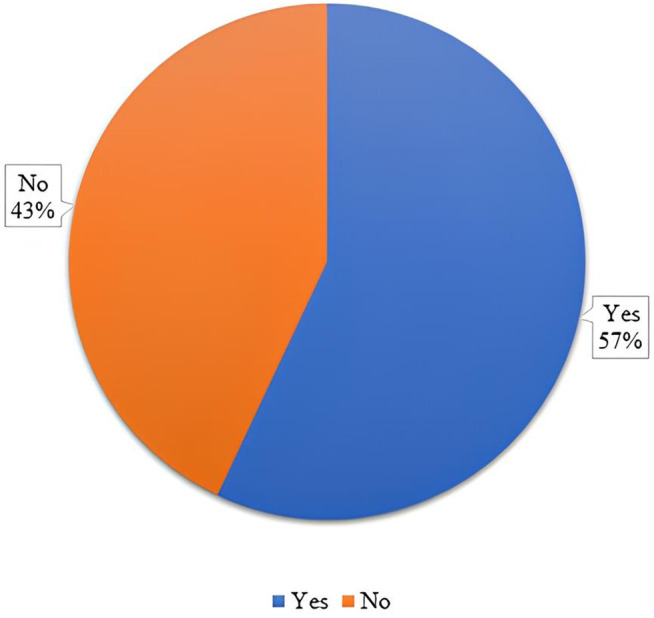
Percentage of participants that reported changes in internet searches during the crisis.

**Fig 8:**
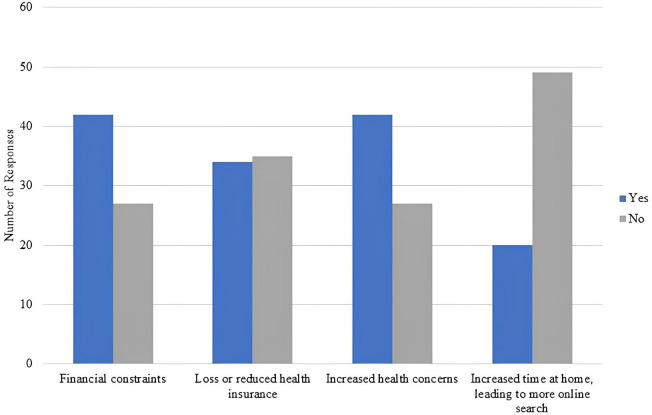
Reasons for increased online health seeking behavior following the 2019 crisis.

Looking at online health seeking behavior during the 2019 crisis in Lebanon, Figs 7 and 8 show that 57% of participants used the internet more during the crisis, mainly due to financial constraints, loss of health insurance, increased health concerns during this phase, and more availability at home to conducts searches.

### Qualitative findings

In total, 13 IDIs were conducted with participants purposively selected from the survey results (refer to materials and methods section). The average duration of the IDIs was 30 minutes and data collection continued until data saturation was achieved. The sample included seven males and six females, with a mean age of 40 years (range: 24–58). Two participants had basic or school-level education, while eleven had attained higher education. Ten participants had sufficient eHEALS scores, whereas three had limited scores. Regarding sources of health information, five primarily relied on their healthcare provider, while eight relied on the internet (Table 9). The overarching themes and subthemes generated are presented in Table 10.

**Table 10:** Themes and subthemes from the qualitative in-depth interviews.

Theme	Subtheme
1. Choosing Between the Internet & the Healthcare Provider: WebMD or MD?	1.1. The internet as a fast primary reference
	1.2. My Doctor Said: “Don’t Google It”
2. Negative Experiences with Internet Searches	2.1. Fear and panic during online health searches
	2.2 How diagnosis sparked curiosity
	2.3. From no information at all to too much information
3. Perceived Usefulness of E-health	3.1. The benefits of the internet over the healthcare provider
	3.2. Animations and videos for better engagement
4. Current Online Practices & Behaviors	4.1. The go to resources online
	4.2. How to check for credibility
	4.3. The role of AI
5. Online Social Support	5.1. Sharing struggles or a preference for ignorance
	5.2. Lack of well-established MS support groups
6. E-health Literacy Challenges	6.1 Individual Challenges
	6.2 External Challenges
7. Internet Surfing During Lebanon’s 2019 Crises	7.1 Online Health Information Seeking Amid Medication Shortages
	7.2 Increased Online Engagement and Health Curiosity During Lockdown
8. What’s Missing and How to Improve	8.1. Unanswered questions and missing research
	8.2. MS still viewed as taboo

**Table 9:** Interview Participants’ Characteristics.

Characteristics	Number (n)
**Gender**	
Male	7
Female	6
Age (Average)	40 years
Average (Range)	24-58 years
**Education**	
Basic or School-level education	2
Higher education	11
**eHEALS Category**	
Limited	3
Sufficient	10
**Primary Source of Health Information**	
Healthcare Provider	5
Internet	8

### 1. Choosing Between the Internet & the Healthcare Provider: WebMD or MD?

#### 1.1. The internet as a fast primary reference.

Consistent with the quantitative results, most participants expressed that the internet is the first resource they seek when looking for health-related information, mainly because it is the fastest and most efficient way. This was especially the case for one participant who lives in a remote area, with limited access to healthcare providers or facilities, leaving the internet as the only resource for them to use.

Participants further explained that with digitization and easy access to information via cellphones, their immediate response when seeking health information is to conduct an internet search. However, the internet is not the only reference these participants relied on, especially in critical situations. Due to the lack of trust in the information and resources online, participants almost always go back to their doctor to validate the information they read, especially when they are experiencing alarming symptoms. One limitation to this is that their healthcare provider is not always available to answer instantly, leaving internet searches as the only resort in such cases. Participants said that they do not have full trust in the information they access online when taking decisions related to medication use or emergency situations. The only time participants expressed confidence to use online information is when it comes to minor concepts such as diet recommendations and general knowledge related to MS (e.g., common symptoms).

Only a few participants stated that their doctor is the first and only reference they seek whenever they want to receive health information. This was the case for participants who have no trust at all in the information available online. However, this lack of trust in online health information can be an issue for participants who need to travel distances to reach their physician and receive proper counseling.

#### 1.2. My Doctor Said: “Don’t Google It”.

Three participants said that their doctor explicitly recommended they do not use the internet to search for information online, mainly to avoid misinformation and to avoid back and forth arguments between Google and the physician. Regardless, these participants continue to use the internet regularly claiming that their “curiosity” is too strong to resist and that they are in constant need of seeking health information related to their condition.

**Quote 1:**

*“When I was first diagnosed with MS the first thing the doctor told me was: whatever happens with you contact me and I will tell you everything you need to know. But my curiosity makes me go on Google and read information related to MS.” — Female, Lebanese, Age 44*

### 2. Negative Experiences with Internet Searches

#### 2.1. Fear and panic during online health searches.

Almost all participants expressed how exaggerated and triggering information related to MS is presented online and on social media. This was a major issue when participants first received their diagnosis and went online to find more information about their condition. Participants expressed that this was a very stressful experience and they either went into a state of denial and stopped searching for MS online or anxiously continued their search on every website they find. The negative information on MS they encountered online were: complete paralysis, bedrest, lack of productivity, gradual deterioration, and death. Participants said that this fear persisted for a while, until eventually some self-taught their way on how to avoid negative information online and how to deal with it as well.

**Quote 2:**

*“For example, the first thing I wanted to know was what MS is, so I searched on YouTube. The first video that came up showed a man lying stiff in bed, completely unresponsive. That was it for me, I couldn’t watch any further” — Male, Lebanese, Age 38*

#### 2.2. How diagnosis sparked curiosity.

Most participants explained that their diagnosis sparked their curiosity in seeking health information online. Meaning, the internet was used by all MS patients interviewed at some point in their MS journey. This was especially true at the beginning, as they had no one to refer to and no prior information about their disease.

**Quote 3:**

*“I never thought to search online about a disease or anything related to symptoms, when I first got diagnosed with MS, that’s when the search started. My siblings taught me how, I used to see how they searched online and I started doing like them.”* *— Male, Lebanese, Age 31*

#### 2.3. From no information at all to too much information.

Participants who got diagnosed a long time ago (over 10 years ago) explained that during that time, not even the internet had information related to MS. The information available online was minimal, vague, and unhelpful. However, they also noted that the current abundance of information is not entirely beneficial, as it can be misleading and contradictory, leaving MS patients in a loop of uncertainty.

**Quote 4:**

*“When I first got diagnosed, it was around 2005, there wasn’t this information that is available like now. Now we’re in 2024 there is a lot of information to the point that it’s confusing.” — Male, Lebanese, Age 59*

### 3. Perceived Usefulness of E-health

#### 3.1. The benefits of the internet over the healthcare provider.

Participants who view the internet as a useful source for health information mentioned several advantages from their perspectives, including fast access to information, not having to pay for consultations, and the abundance of information. This was particularly important when no one around them was able to answer their questions and the lack of awareness on MS. They further explained that using the internet is in some cases more appropriate than reaching out to their healthcare provider due to late night questions that they might have, questions that may seem too simple to ask a professional, and a need for rich references related to medication side effects. The internet can also provide participants more details and explanations than a professional can during consultations due to time constraints. Additionally, online resources often include visual representations that enhance understanding.

One participant stated that the internet is a source for MS patients to have autonomy and take decisions that best fit their needs and improve their quality of life (QOL), which is a necessity for people with MS. However, some participants also explained that the internet can only be helpful if used properly. When people with MS utilize and process the information correctly then it can be very helpful, or else it can be harmful.

**Quote 5:**

*“Because of what I read online I stopped smoking. I read online that smoking can push you back by 50%, but quitting will make you feel much better. My doctor only told me to stop smoking, but he did not explain to me why.” — Female, Lebanese, Age 56*

#### 3.2. Animations and videos for better engagement.

Similar to the quantitative results, all participants interviewed indicated that visuals and animations with voiceovers were the most preferred formats when accessing health information online. Participants explained that due to MS, they have shorter attention spans, find it difficult to read for extended periods, and are more prone to forgetfulness. Which is why videos and animations are the most effective and engaging way for them to receive health information online.

### 4. Current Online Practices & Behaviors

#### 4.1. The got to resources online.

Only two participants were able to identify specific MS websites that they resort to during their online searches, including international MS organizations such as: the Multiple Sclerosis International Federation (MSIF), MS Canda, MS USA etc. To these participants, MSIF serves as an information platform that can be used for every MS topic that patients or their caregivers might need, including information in Arabic. The remaining participants were unaware of these resources.

On the other hand, other participants said they do not have a specific online resource they resort to, saying “every time I go somewhere new”. Google remains the most prevalently used search engine, followed by YouTube, and occasionally Facebook. The online pages of Lebanese MS NGOs also serve as a resource for patients, offering access to patient testimonies, workshops, events, and the latest updates on the situation in the country, including drug shortages.

#### 4.2. How to check for credibility.

None of the participants were completely confident in their methods for verifying the credibility of information they read online, primarily relying on their “logic” and having to confirm with their healthcare provider. Participants repetitively explained that after reading health information online, self judgement and logic are used to assess credibility, which is something participants feel they have acquired over the years from “experience”. This is coupled with participants using multiple references in their searches and relying on the most frequently repeated answer. Again, consulting their doctor is a method participants use to feel more confident about the information they access and read online.

Another method participants sometimes use is to make sure they read credible information posted only by well-known and recognized universities, hospitals, and MS organizations. They also occasionally verify the author’s background.

**Quote 6:**

*“I don’t know how to distinguish, I read everything and I might believe something that is not true. But I read everything. It’s difficult for me to distinguish between credible and non-credible information.” — Male, Lebanese, Age 31*

#### 4.3. The role of AI.

There were two perspectives on the new role of AI and E-health literacy by participants who are aware of this tool. Two participants said that if used properly, feeding the AI tool with the right information can be used to quickly access and analyze health information related to MS. Another participant stated that she uses AI during her daily searches for the quickest and most collective information, however she does not trust its credibility completely, it only guides her thinking and interest.

However, two other participants explained that at this stage, AI is not ready to provide MS patients with guidance on health information, claiming that it is still new and might give faulty information, even though they consider it helpful with their jobs.

**Quote 7:**

*“It [AI] enlightens me, instead of me searching here and there it gives me the information instantly. But I cannot trust it 100%.” — Female, Lebanese, Age 56*

### 5. Online Social Support

#### 5.1. Sharing struggles or a preference for ignorance.

Most participants expressed that one of the most important benefits of online health seeking information, is finding other MS patients to talk to and share experiences with. To these participants, the main role of social media platforms is to connect MS patients together for both emotional and informational support. Participants emphasized the importance of knowing they are not alone in their struggles and highlighted the value of mutual support, particularly through sharing information about medication availability in the country during shortages. Additionally, it was through these platforms that MS patients were able to find local NGOs in the country that can further support them financially and guide them throughout their MS journey.

**Quote 8:**

*“No one understands you except those that are like you. That’s the importance of talking to people that are feeling the same as you, knowing that every person’s case is different.” — Female, Lebanese, Age 56*

However, two participants had opposing views, noting that because MS is such a varied disease, it might not always be beneficial to listen to what others have been through, especially that not everyone will reach advanced stages in their MS journey. These participants explained that sometimes it’s better to not know how severe the disease can get, for a more relaxed mind.

**Quote 9:**

*“They put me in a WhatsApp group for MS and I received on it messages from MS patients in more advanced stages than me, so things start to take a toll at you, that maybe I will one day reach this stage.” — Female, Lebanese, Age 31*

#### 5.2. Lack of well-established MS support groups.

Two participants noted that while social media groups and connections through local NGOs offer opportunities for MS patients to connect, the country lacks well-established and professional support groups. According to these participants, who have sought to connect with other MS patients for support, the absence of official support groups has led to a generally negative experience. They emphasized that this is a significant gap in support that needs to be addressed.

**Quote 10:**

*“I went to a support group for diseases related to the nervous system but everyone there was older in age, and this made me more uncomfortable. So I believe if there is a support group for all of us in our 20s, this will encourage me more. I went and I’m 24 but everyone there was above 70 years so I started looking and thinking will I end up like this? It was very traumatizing.” — Female, Lebanese, Age 24*

### 6. E-health literacy challenges

#### 6.1. Individual challenges.

A few participants reported no perceived challenges when accessing health information online, from their own perceptions and judgment. However, other participants identified language barriers as an obstacle they face, including the lack of information available in Arabic and the presence of technically complicated words. Participants also mentioned that they did not have any guidance when surfing health information online.

#### 6.2. External challenges.

The most reported challenges by participants were: the contradicting information that is available online, the variability of MS as a disease, and the slow internet connections. “No case is like the other” is a phrase that was repeated by participants, explaining that not everything they read online can be applied to their specific condition.

**Quote 11:**

*“I need something tailored to me because each MS case is unique, with its own exercises, medications, and way of living. No case is like the other.” — Female, Lebanese, Age 56*

### 7. Internet Surfing During Lebanon’s 2019 Crises

#### 7.1. Online health Information seeking amid medication shortages.

The most reported use of the internet during Lebanon’s 2019 crisis was related to medication shortages. Participants mostly went online to search for information on the availability of medications, the side effects of the new treatment they shifted to using, and complementary therapies they might use beyond medicine. During this time participants were also looking for information on what will happen if they miss a dose or stop their medication completely due to shortages in the country. Connecting online with other MS patients and local NGOs was also mentioned by participants to get further updates during the crisis.

**Quote 12:**

*“I switched my medication during the crisis, it was a very hard time we didn’t understand what was happening and it was very confusing. I didn’t know why my doctor switched my medication so I had to do more internet searches during that time.” — Male, Lebanese, Age 59*

#### 7.2. Increased Online Engagement and Health Curiosity During Lockdown.

Additionally, during lockdown, participants said they had more time to spend online and to surf the web, which increased their curiosity and caused them to engage in more online health seeking behaviors.

### 8. What’s Missing and How to Improve

#### 8.1. Unanswered questions and missing research.

When asked about what remains unanswered during participants’ online health seeking behaviors, almost all agreed that the absence of a cure and consensus about the causes of MS is something that is still missing. This leaves patients wondering what will eventually happen to them, which is something they try to answer repeatedly. Also, the information that is available online cannot always be adopted to the context of Lebanon, including certain lifestyle recommendations that are unaffordable or structurally difficult to perform.

**Quote 13:**

*“For example, biking with MS is good but if you go biking on the road in Lebanon, you stress out. Even if jogging and exercise is very good, but the mode of transportation in this country is by car not walking.” — Male, Lebanese, Age 35*

Several participants also mentioned that they do not hear of any local research or clinical trials happening in the country and they were never invited to be a part of it. Even on both local and international levels, information related to research on MS is neither transparent nor easily accessible when they seek it.

#### 8.2. MS still viewed as taboo.

One idea that all participants emphasized on was how MS is still considered a taboo in Lebanon, with people with MS ashamed of their condition and people around them having very little knowledge on the disease. Participants voiced out that they want MS to be seen as any other chronic condition in society, without having to feel like an outsider. Also, they believe that there is a huge gap in the awareness of their community on MS.

**Quote 14:**

*“I haven’t told anyone and no one knows I have MS except for the people that are very very close to me. In university none of my classmates know and three quarters of my family members don’t know either because the information that is available on MS is negative 98% of the time and can destroy a person’s life [MS portrayed negatively online]. So I think there is a gap in information that makes MS more down to earth or closer to what is actually happening to these patients.” — Female, Lebanese, Age 24*

## Discussion

This is the first study to evaluate the e-health literacy levels of MS patients in Lebanon. Through a mixed methods approach, the results of the quantitative survey were further explained using IDIs to dive deeper into the experiences of this population and their online health seeking behaviors. eHEALS scores showed that 45 (31.5%) MS participants had limited scores and 98 (68.5%) MS participants had sufficient eHEALS scores. This indicates that most MS participants had a sufficient level of e-health literacy. Notably, MS participants with sufficient eHealth literacy (median score ≥ 27) were more likely to perceive the internet as an important source of health information and expressed greater confidence in identifying biased or misleading content online compared to those with limited e-Health literacy. Interestingly, language and age also appeared to play a role in shaping online behaviors, with employed participants more likely to complete the survey in English and older participants reported spending less time online each day. Although previous studies have identified an association between educational status and e-health literacy [28,29], no such relationship was observed in our sample potentially due to the limited sample size. Compared to a 2019 study on general internet users in Lebanon, the eHealth literacy levels of MS patients were very similar to those of the general internet-using population in Lebanon (mean (SD) eHEALS score: 28.79 (5.51)). By cautiously interpreting these results, this suggests that individuals living with MS in Lebanon possess similar levels of e-health literacy as general internet users.

Consistent with other e-health literacy studies, MS patients in Lebanon actively seek health information online, relying primarily on search engines such as Google [24,30,31]. However, despite achieving above-average scores on the eHEALS questionnaire, survey responses and qualitative interviews revealed that most participants have concerns about the credibility of the content they read online, especially when this information is encountered on social media platforms. Rather than using standardized methods to assess the accuracy and credibility of this information, many participants reported relying on “logic” and “personal experience”. The unregulated nature of internet results thus places MS patients at risk of misinformation and misleading or inaccurate health information.

Multiple sclerosis patients encounter several barriers that complicate their access to credible and understandable online resources. By incorporating all six domains of the Lily model into our data collection tools and asking questions related to traditional, information, media, scientific, health, and computer literacy [4], we were able to gain a comprehensive understanding of the potential barriers participants may face in each domain. The most commonly reported challenges by MS participants included: information overload, lack of trust and credibility in online information, complex medical terms, lack of MS-specific information, conflicting information, absence of Arabic resources, and non-user-friendly websites. These challenges can be mapped under the: traditional, informational, scientific, and media literacy domains of the Lily Model. Lastly, participants expressed a preference for accessing information online through pictures and visual illustrations, noting that this approach enhances their understanding of complex concepts and reduces potential challenges.

Amidst the 2019 crisis in Lebanon, most participants did use the internet for information related to MS, but it was more to communicate and gather information on the availability of their medication. During this time, when participants were forced to switch treatments because of shortages, they had to look for information on their new medication, its side effects, and the impact of missing or stopping their medication. Based on the quantitative findings, this increase in internet search during the crisis was largely driven by financial constraints, heightened health concerns, and increased time at home due to lockdowns and roadblocks.

E-health literacy is a growing area of interest when it comes to health promotion and health related behaviors. Although a meta-analysis found a moderate positive link between eHealth literacy and health behaviors, our findings suggest that MS patients’ initial experiences with online health information may hinder their online health seeking behavior and discourage the development of their eHealth literacy skills [32]. This stems from the negative and triggering information that is present online, leading MS patients to sometimes avoid online health seeking behavior completely and impacting their journey from e-health literacy →to self-efficacy→ to self-care ability→ to health promotion behaviors [33]. Participants also reported experiencing significant emotional distress during their online health searches, often leading to stress, anxiety, and fear. This is where the timeliness of accessing positive health information and health promotion interventions are important, especially upon diagnosis, a critical period that can shape subsequent information-seeking behaviors [34].

Several recommendations can be made to enhance the experience of individuals living with MS in Lebanon when navigating health information online. It is critically important to support MS patients from the point of diagnosis on how seeking health information online can empower their journey with the disease. This involves providing them with the skills, resources, and self-efficacy needed to access, read, understand, and make decisions that improve their health using online resources. As local MS patient organization in the country are viewed as trusted resources to this population, with active programs and participation with MS patients, e-health literacy should be a focus point of these organizations to ensure patients avoid fear and panic upon and during diagnosis. Interventions should focus on providing this population with available and highly credible online resources and platforms in multiple languages, such as those offered by MSIF. This should also include sharing up-to-date research advances on their pages and establishing a professional online support group for patients to connect and share experiences together. There is a clear need for establishing a formal online social support group, ensuring that participants can voluntarily attend and communicate with other MS patients. Additionally, participants expressed a strong desire for increased awareness and destigmatization of MS through the support of community education campaigns led by NGOs, academic institutions, and public health authorities. Finally, healthcare providers also have a role in empowering patients to take an active role in their disease journey and by providing them with oral and written information on MS upon diagnosis, including online reliable resources [35].

This study offers several notable strengths, it is the first study to evaluate e-health literacy levels among MS patients in Lebanon, addressing a significant gap in both regional and global literature. Few studies worldwide have examined e-health literacy within a specific clinical population, making this research particularly unique and relevant. Second, it is among the few to fully integrate all six components of the Norman and Skinner e-health literacy framework, a comprehensive approach often overlooked in digital health interventions. Third, rather than focusing solely on general internet skills, this study delves into the specific online sources MS patients use and trust for health information, offering deeper insight into their behaviors and preferences. Lastly, this study provides targeted, user-driven recommendations to enhance e-health literacy, grounded in the actual needs and perspectives of the participants.

This study is subject to several limitations that should be acknowledged. First, due to the purposive sampling strategy, the findings cannot be generalized to the broader population of MS patients in Lebanon. Second, the cross-sectional design limits the ability to infer causal relationships between variables. One aspect of MS that was missing and not asked in the survey was details into participants’ diagnosis and prognosis, meaning when they were diagnosed with MS and how progressive their disease is, which may have generated further insights. Response bias may be a concern in this study, as the eHEALS measures how participants perceive their e-health literacy rather than their actual skills, and the interviews also relied on self-reported information.

## Conclusion

This study represents the first evaluation of e-health literacy level among MS patients in Lebanon, highlighting a significant reliance on the internet for health information. Despite the moderate e-health literacy score identified, participants expressed concerns about the accuracy and emotional impact of online information. This highlights the need to raise awareness among this population on how to assess the credibility of online information and identify high-quality resources to better support their disease management journeys.

## Supporting information

S1 Data Set Dataset. Minimal Data Set.
(XLSX)

S1 Table Responses to eHEALS items by Likert scale levels (n, %). (DOCX)

S1 File Final Interview Guide. (DOCX)

S2 File Initial Interview Guide. (DOCX)
