# Supplementary material for: E-health literacy levels of multiple sclerosis patients in Lebanon
Source: PLoS One. 2025 Oct 31;20(10):e0335084. doi: 10.1371/journal.pone.0335084 (PMC12578138; doi:10.1371/journal.pone.0335084)
Supplement: S1 Table — (DOCX) [file pone.0335084.s002.docx]

**S1 Table. Responses to eHEALS items by Likert scale levels (n, %)**

| **eHEALS items** | **Strongly Disagree**  **n (%)** | **Disagree**  **n (%)** | **Neutral**  **n (%)** | **Agree**  **n (%)** | **Strongly Agree**  **n (%)** |
| --- | --- | --- | --- | --- | --- |
| **I know what health resources are available on the Internet.** | 4 (2.80) | 13 (9.09) | 62 (43.36) | 54 (37.76) | 10 (6.99) |
| **I know where to find helpful health resources on the Internet.** | 5 (3.50) | 4 (2.80) | 47 (32.87) | 76 (53.15) | 11 (7.69) |
| **I know how to find helpful health resources on the Internet.** | 4 (2.80) | 4 (2.80) | 47 (32.87) | 78 (54.55) | 10 (6.99) |
| **I know how to use the Internet to answer my health questions.** | 4 (2.80) | 4 (2.80) | 26 (18.18) | 98 (68.53) | 11 (7.69) |
| **I know how to use the health information I find on the Internet to help me.** | 3 (2.10) | 3 (2.10) | 28 (19.58) | 97 (67.83) | 12 (8.39) |
| **I have the skills I need to evaluate the health resources I find on the Internet.** | 4 (2.80) | 12 (8.39) | 39 (27.27) | 79 (55.2) | 9 (6.29) |
| **I can tell high-quality health resources from low-quality health resources on the Internet.** | 6 (4.20) | 8 (5.59) | 41 (28.67) | 76 (53.15) | 12 (8.39) |
| **I feel confident in using information from the Internet to make health decisions.** | 12 (8.39) | 10 (6.99) | 42 (29.37) | 70 (48.95) | 9 (6.29) |
