# Supplementary material for: E-health literacy levels of multiple sclerosis patients in Lebanon
Source: PLoS One. 2025 Oct 31;20(10):e0335084. doi: 10.1371/journal.pone.0335084 (PMC12578138; doi:10.1371/journal.pone.0335084)
Supplement: S1 File — (DOCX) [file pone.0335084.s003.docx]

**IDI Guide – English - Initial**

1. When you have a question related to MS, where do you usually go to find information and how? *(Probe on search strategies and process, what information do you usually look for related to your health, etc.)*
2. How easy is it for you to distinguish between credible and non-credible information posted online?
3. Sometimes websites say different things about the same topic. What do you do when that happens?
4. What type of message formats do you prefer when accessing information online related to your condition?
5. How do you feel about the degree of representation and relevance of online health information in relation to your personal experiences and concerns as an MS patient *(meaning do you feel it is tailored to your specific needs as an MS patient and represents your needs and concerns)*?
6. What MS-related tools or platforms do you wish were easier to find online?
7. How do you think online health information platforms can better serve the needs of MS patients like yourself?
8. Is there anything else you'd like to share or think we should know about e-health literacy and MS?
