# Supplementary material for: E-health literacy levels of multiple sclerosis patients in Lebanon
Source: PLoS One. 2025 Oct 31;20(10):e0335084. doi: 10.1371/journal.pone.0335084 (PMC12578138; doi:10.1371/journal.pone.0335084)
Supplement: S2 File — (DOCX) [file pone.0335084.s004.docx]

**IDI Guide – English - Final**

1. Can you describe the last time you looked for health information online? *(Probe on search strategies and process, what information do you usually look for related to your health)*
2. How do you distinguish between credible and non-credible information posted online?
3. How do you handle conflicting information that you encounter online while doing your search using different sources?
4. What type of message formats do you prefer when accessing information online related to your condition *(ex: videos, audios, written text, etc…)*?
5. How do you feel about the degree of representation and relevance of online health information in relation to your personal experiences and concerns as an MS patient *(meaning do you feel it is tailored to your specific needs as an MS patient and represents your needs and concerns)*?
6. What resources or tools related to MS do you feel are lacking or could be more accessible online *(Ex: Informational Websites and Databases, Support and Community Platforms, Telemedicine and Online Consultations, Mobile Applications, Exercise and Physical Activity Resources)*?
7. How do you think online health information platforms can better serve the needs of MS patients like yourself?
8. Is there anything else you'd like to share or think we should know about e-health literacy and MS?
